# Y-Shaped Bilateral Self-Expandable Metallic Stent Placement for Malignant Hilar Biliary Obstruction: Data from a Referral Center for Palliative Care

**DOI:** 10.1155/2014/151502

**Published:** 2014-03-23

**Authors:** R. Di Mitri, F. Mocciaro

**Affiliations:** ^1^Gastroenterology and Endoscopy Unit, A.R.N.A.S. Civico-Di Cristina-Benfratelli Hospital, Piazza Leotta No. 4, 90100 Palermo, Italy; ^2^Via Strada Ferrata 44/D, 90046 Monreale, Italy

## Abstract

*Background and Aim*. Malignant hilar strictures are a clinical challenge because of the current therapeutic approach and the poor prognosis. In recent years, self-expandable metallic stents have proven more effective than plastic stents for palliation of malignant hilar strictures, with the bilateral stent-in-stent technique registering a high success rate. We report our experience with Y-shaped endoscopic self-expandable metallic stents placement for treatment of advanced malignant hilar strictures. *Methods*. From April 2009 to August 2012, we prospectively collected data on patients treated with Y-shaped SEMS placement for advanced malignant hilar carcinoma. Data on technical success, clinical success, and complications were collected. *Results*. Twenty patients (9 males) were treated (mean age 64.2 ± 15.3 years). The grade of malignant hilar strictures according to the Bismuth classification was II in 5 patients (25%), IIIa in 1 (5%), and IV in 14 (70%). The mean bilirubin level was 14.7 ± 4.9 mg/dL. Technical success was achieved in all patients, with a significant reduction in bilirubin levels (2.9 ± 1.7 mg/dL). One patient experienced cholangitis as early complication, while in 2 patients stent ingrowth was observed. No stents migration was recorded. There was no procedure-related mortality. At the end of the follow-up (7.1 ± 3.1 months), 13 of the 20 patients (65%) had died. *Conclusions*. Our experience confirms endoscopic bilateral self-expandable metallic stents placement with stent-in-stent technique (Y-shaped configuration) as a feasible, effective, and safe procedure for palliation of unresectable malignant hilar strictures.

## 1. Introduction

Malignant hilar strictures (MHS), type ≥2 according to the Bismuth-Corlette classification of cancers of the human biliary tract [1], are a clinical challenge because of the current therapeutic approach (surgical or nonsurgical) [2] and the poor prognosis associated with this form of cancer. Cholangiocarcinoma is the leading cause of all MHS which require, in inoperable patients, a conservative approach only with stent placement through percutaneous transhepatic cholangiography or endoscopic retrograde cholangiopancreatography (ERCP) [3]. Despite the fact that multiple plastic stenting is a feasible and valid treatment, self-expandable metallic stents (SEMS) have proven to be more effective than plastic stents for hilar tumor palliation [4], with bilateral stent-in-stent placement registering a high success rate [5]. In the current study, we report our experience on feasibility, efficacy, and safety of Y-shaped endoscopic SEMS placement in advanced malignant hilar carcinoma.

## 2. Methods

From April 2009 to August 2012, we prospectively collected data on patients treated with Y-shaped SEMS placement for advanced malignant hilar carcinoma. All patients were diagnosed as having MHS by computer-assisted tomography or magnetic resonance imaging. Histologic and cytologic confirmation of malignancy was made for all the patients. Indication for stent placement was an increase in bilirubin levels with evidence of intrahepatic bile ducts dilatation. Patients were treated within 48 hours from the admittance. All patients were not treated previously. Data were collected in an electronic database and subsequently exported to the statistical software for final analysis.

**Figure 1:**
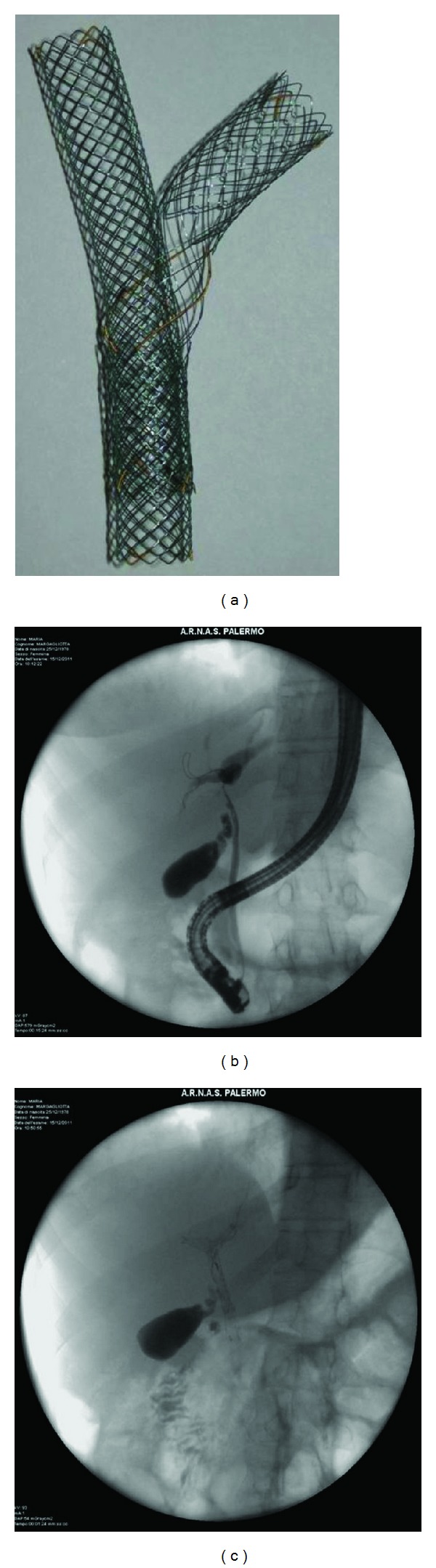
(a) Niti-S stent assembled (Y-shaped configuration). (b-c) Images from a patient with hilar cholangiocarcinoma before (b) and after (c) stent placement.

All patients were hospitalized and complete full blood count, chemistry, and coagulation parameters were obtained. Stent placement was discussed with the surgeon and both tumor characteristics/location and overall clinical condition were taken into account. An informed consent was always obtained before the procedure. All endoscopic procedures were performed with a therapeutic channel video duodenoscope with the patient under deep sedation with propofol. SEMS (Niti-S Biliary Y stent, Taewoong, Seoul, Republic of Korea) were placed during ERCP and according to manufacturer's instructions, by an experienced endoscopist. The length and the size of SEMS were the same in all treated patients. After common bile duct cannulation, cholangiography, and subsequent sphincterotomy, a guidewire was placed, under fluoroscopic guidance, across the left hepatic duct. Evaluation of the severity of the stricture was made with contrast injection. The first uncovered SEMS, with a wide-open central mesh were placed across the hilar stricture. If needed, balloon dilation was performed before stent placement. The guidewire was then withdrawn slowly and, with the aid of a 5.5 French catheter, inserted under fluoroscopic guidance into the wide-open central mesh of the first stent, identified by radiopaque markers. The second uncovered SEMS were placed through the central crossed mesh of the primary stent (Y-shaped configuration) to drain the right hepatic duct (Figure 1).

All patients, data on technical success, clinical success, and complications were collected. All patients were followed up in the outpatient clinic or by phone until death.

Technical success was defined as successful bilateral SEMS placement across the stricture, confirmed by radiological markers at fluoroscopy, with outflow of contrast medium and/or bile through the stents. Clinical success was defined as reduction of bilirubin levels of at least 75% of the pretreatment value within the first month. Complications were defined as any event related to SEMS placement (included occlusion). Complications were defined as early if complications occurred within 30 days and late if occurring after 30 days. We also considered any bleeding due to endoscopic sphincterotomy, according to the Cotton criteria [6], as a complication. Stent occlusion was defined as recurrence of jaundice or significant increase in bilirubin level with concomitant US or CT evidence of intrahepatic bile duct dilatation that required an endoscopic, percutaneous, or surgical procedure. Stent patency was defined as the period of time between insertion and stent occlusion. Each death was investigated as to whether it was related to the SEMS placement or to the natural history of the underlying malignancy.

Data were analyzed using the software package SPSS 15 (SPSS Inc., Chicago, IL, USA). Continuous variables were summarized as mean (±standard deviation [SD]) or median (range) according to their distribution. Categorical variables were summarized as frequency and percentage.

## 3. Results

We enrolled 20 patients, 9 males (45%) and 11 female (55%), with a mean age of 64.2 ± 15.3 years. The causes of hilar biliary obstruction were cholangiocarcinoma in 10 patients (50%), metastatic colon cancer in 5 (25%), metastatic pancreatic cancer in 3 (15%), and hepatocarcinoma in 2 (10%). The types of MHS according to the Bismuth classification were II in 5 patients (25%), IIIa in 1 patient (5%), and IV in 14 patients (70%). The mean bilirubin level was 14.7 ± 4.9 mg/dL. The diagnosis of malignancy was confirmed with tissue samples (brushing or forceps biopsy). Technical success was achieved in all patients. Eleven of the 20 patients (55%) were treated with balloon dilation before stent placement. After the procedures, a significant reduction in bilirubin levels was observed in all treated patients, with a mean bilirubin level at discharge of 2.6 ± 1.5 mg/dL. No patients received chemotherapy after SEMS placement. The median stent patency was 7 months (range 3–13), while mean survival time was 7.1 ± 3.1 months. No differences of SEMS patency were observed among the II, III, and IV type of MHS. At the end of the follow-up, 13 of the 20 patients (65%) had died of tumor progression.

## 4. Adverse Events

One patient (5%) experienced cholangitis as an early complication, which resolved with medical therapy. Among the late complications, 2 patients (10%) were treated with plastic stent placement (10 French) through the metallic stent because of SEMS ingrowths at 3 and 10 months, respectively. The “Y-shape” did not cause trouble to insert the plastic stents but the stents were placed on a wire guide previously inserted in the occluded SEMS. There was no procedure-related mortality.

## 5. Discussion

Endoscopic insertion of plastic endoprosthesis has become widespread in patients with malignant proximal biliary obstruction, limiting surgical intervention to a minority of selected patients in whom the tumor seems to be resectable [7]. Unfortunately, despite technological improvements and long-term antibiotic prophylaxis [8, 9], blockage of plastic stents cannot be prevented indefinitely.

The diffusion of SEMS has modified the palliative approach in unresectable patients. A recent randomized controlled trial [10] on SEMS versus plastic stent placement showed a higher 6-month patency rate for SEMS (81% versus 20%; *P* = 0.0012), with no significant difference in terms of overall survival time. In addition, the mean number of reinterventions because of stent failure was lower in the SEMS group (0.63 versus 1.80 times/patient; *P* = 0.0008). The total cost of the treatment was significantly lower in the SEMS group than in the plastic-stent group (*P* = 0.022).

Drainage of obstructed ductal systems has been strongly advocated as allowing for a significant reduction in morbidity and mortality because of better bile flow [11]. Though technically demanding, bilateral placement of two parallel metal expandable endoprosthesis has proven to be a safe and feasible procedure, with a high success rate and little need for further biliary reintervention in patients with unresectable hilar cholangiocarcinoma. Cheng et al. [12] reported, in 36 patients with Bismuth type I/II-III-IV lesions, an insertion success rate of 97%, with only 14% of complications at 30 days. Furthermore, mean stent patency was 169 days, with a rate of stent occlusion at the end of the follow-up of 31%. In 2009, Naitoh et al. confirmed the feasibility of endoscopic bilateral SEMS placement (90% insertion rate success), also showing that this approach was more effective than unilateral drainage in terms of cumulative stent patency (488 versus 210 days, *P* = 0.009), especially in cases of cholangiocarcinoma, with the same risk of early/late complications and cumulative survival time [13].

In order to improve the feasibility of bilateral SEMS placement, and to avoid the parallel placement of two stents, Silverman and Slivka [14] presented a new technique, in 1996, in which a stent (Wallstent, Boston Scientific, Natick, MA, USA) was placed through the fenestration of SEMS, obtained with 7 French Soehendra stent extractor, previously placed in the right intrahepatic bile duct. In 2007, Lee et al. [15] carried out a pilot study on a newly designed Y metal stent using a biliary stent with wide-open central mesh first (Niti-S Biliary Y stent; Taewoong, Seoul, Republic of Korea), followed by a second SEMS placement (Niti-S or Niti-D stent, resp.; Taewoong) in the contralateral hepatic duct through the central open mesh of the other stent. The authors reported technical success in 8 of the 10 patients (80%), with a functional success rate of 100% among patients in whom bilateral stents were successfully placed by endoscopy. The early complication rate was 0%, with an occlusion rate of 25% and a median of stent patency period of 217 days.

Since then, several studies have reported similar experiences in larger number of patients treated with the stent-in-stent technique [16–24] showing technical success rate between 85 and 100% and functional success rate between 95 and 100% (Table 1). As reported by Naitoh et al. [23], stent-in-stent technique is to be preferred instead of side-by-side deployment in terms of complications (*P* = 0.016) despite the fact that there were no significant intergroup differences in technical and functional success between the two technique.

An interesting historical control study compared 20 patients with unresectable malignant hilar biliary obstruction who had undergone endoscopic bilateral Y-configured biliary drainage with SEMS placement to 37 patients who had undergone bilateral drainage with plastic stents (control group) [20]. At the end of the study, the technical success rate in the SEMS group was 100% (lack of data from control group). The success rate of biliary decompression was 95% versus 89% (*P* = 0.65). In the follow-up period, the incidence of stent occlusion was significantly lower in the SEMS group than in the other group (30% versus 62%, *P* = 0.028), with a mean stent patency of 250 days versus 115 days (*P* = 0.0061).

A recent large retrospective study by Kim et al. [24], on placement of the newly designed Y-stent with a slimmer open-cell second stent, showed a successful placement and functional success rate of 87% and 97%, respectively.

Although, as reported above, Y-shaped SEMS are effective and safe in MHS treatment, some possible problems should be discussed: (1) insufficient opening of the central portion of the first stent which can be solved with balloon dilation, (2) inaccurate release of the central open mesh at the hilar bifurcation which can be improved by closing and repositioning the stent, and (3) stent occlusion which can be treated with balloon extraction (in case of sludge or stones), with biliary drainage through percutaneous transhepatic cholangiography or with new plastic or metallic stent placement, or.

In our study, we had a technical and clinical success rate of 100% using 2 nitinol metallic stents placed with the stent-in-stent technique to obtain the characteristic Y-shaped configuration. A significant biliary drainage was achieved also in all patients with Bismuth IV. The rate of observed complications was low, and this data is in agreement with data from a recent review by Kogure et al. [21] in which no differences in complications were reported between the different types of SEMS.

In conclusion, endoscopic Y-shaped bilateral stent-in-stent SEMS placement is safe and effective for the palliation of unresectable MHS. This is because the technique more closely resembles the physiological bilateral drainage state than does unilateral drainage. These results should be confirmed by larger prospective series and randomized controlled trials so this technique might gain consensus and become a standard of care.

## Figures and Tables

**Table 1:** Studies on bilateral self expandable metallic stent placement in malignant hilar strictures.

Study	Number of patients	Type of SEMS	Technical success	Functional success	Tumor ingrowth or overgrowth	Complications (early/late)	SEMS patency
Lee et al. (2007) [15]	10	Niti-S	80%	100%	25%	0%/0%	217 days
Park et al. (2009) [16]	35	Bonastent	94.3%	100%	0%	0%/0%	150 days
Kim et al. (2009) [17]	34	Niti-S	85.3%	100%	31%	10.3%/37.9%	186 days
Chahal and Baron (2010) [18]	21	Flexxus	100%	—	33.3%	—	189 days
Kogure et al. (2011) [19]	5	Niti-S LGD*	100%	—	40%	20%/—	202 days
Kanno et al. (2011) [20]	20	Niti-S	100%	95%	30%	5%/0%	250 days
Hwang et al. (2011) [22]	30	Niti-S	86.7%	100%	38.5%	10%/0%	176 days
Naitoh et al. (2012) [23]	24	Niti-S	100%	100%	42%	4%/8%	104 days
Kim et al. (2013) [24]	66	Niti-S	87.9%	100%	34.2%	12.1%/55.2%	152 days
Current study	20	Niti-S	100%	100%	10%	5%/10%	210 days

*Large cell D-type.
